# Potential Therapeutic Functions of PU-91 and Quercetin in Personalized Cybrids Derived from Patients with Age-Related Macular Degeneration, Keratoconus, and Glaucoma

**DOI:** 10.3390/antiox12071326

**Published:** 2023-06-22

**Authors:** Nasim Salimiaghdam, Lata Singh, Mithalesh Kumar Singh, Marilyn Chwa, Shari Atilano, Zahra Mohtashami, Anthony Nesburn, Baruch D. Kuppermann, M. Cristina Kenney

**Affiliations:** 1 Gavin Herbert Eye Institute, Director of Mitochondria Research Laboratory, University of California Irvine, 843 Health Science Rd., Hewitt Hall, Room 2028 Irvine, Irvine, CA 92697, USA; salimiag@hs.uci.edu (N.S.); drlata.singh@aiims.edu (L.S.); mithales@hs.uci.edu (M.K.S.); mchwa@hs.uci.edu (M.C.); satilano@hs.uci.edu (S.A.); zmohtash@hs.uci.edu (Z.M.); anesburn@hs.uci.edu (A.N.); bdkupper@hs.uci.edu (B.D.K.); 2 Cedars-Sinai Medical Center, Los Angeles, CA 90048, USA; 3 Department of Pathology and Laboratory Medicine, University of California Irvine, Irvine, CA 92697, USA

**Keywords:** AMD cybrids, Glc cybrids, KC cybrids, PU-91, quercetin, combined PU-91 and quercetin

## Abstract

The aim of this study is to investigate the therapeutic potential of higher doses of PU-91, quercetin, or in combination on transmitochondrial cybrid cell lines with various mtDNA haplogroups derived from patients with age-related macular degeneration (AMD), glaucoma (Glc), keratoconus (KC), and normal (NL) individuals. Cybrids were treated with PU-91 (P) (200 µM) alone, quercetin (Q) (20 µM) alone, or a combination of PU-91 and quercetin (P+Q) for 48 h. Cellular metabolism and the intracellular levels of reactive oxygen species (ROS) were measured by MTT and H2DCFDA assays, respectively. Quantitative real-time PCR was performed to measure the expression levels of genes associated with mitochondrial biogenesis, antioxidant enzymes, inflammation, apoptosis, and senescence pathways. PU-91(P) (i) improves cellular metabolism in AMD cybrids, (ii) decreases ROS production in AMD cybrids, and (iii) downregulates the expression of *LMNB1* in AMD cybrids. Combination treatment of PU-91 plus quercetin (P+Q) (i) improves cellular metabolism in AMD, (ii) induces higher expression levels of *TFAM*, *SOD2*, *IL6*, and *BAX* in AMD cybrids, and (iii) upregulates *CDKN1A* genes expression in all disease cybrids. Our study demonstrated that the P+Q combination improves cellular metabolism and mitochondrial biogenesis in AMD cybrids, but senescence is greatly exacerbated in all cybrids regardless of disease type by the P+Q combined treatment.

## 1. Introduction

Mitochondria are organelles responsible for energy metabolism and ATP production through oxidative phosphorylation [1,2]. It is surrounded by two membranes enclosing a dense matrix of metabolism-related enzymes and a circular genome encompassing thousands of copies of the mitochondrial DNA (mtDNA) [3]. Pathologic mitochondrial metabolism could lead to increased production of reactive oxygen species (ROS) and alteration of transcription factors activities leading to the proliferation of cancer cells [4]. Furthermore, previous studies have established the critical role of mtDNA mutation in tumorigenesis and cancer cell adaptation to environmental changes [5].

A growing body of evidence indicates a remarkable link between the aging process and alterations in mitochondrial function [6]. Lane et al. demonstrated that aging causes mtDNA damage and deterioration of mitochondrial respiratory capacity, resulting in increased levels of ROS [7]. A significant number of recent studies have demonstrated the potential role of mitochondrial changes as an effective factor in a variety of age-related diseases such as age-related macular degeneration, Parkinson’s, and Alzheimer’s diseases [8,9,10]. Our lab previously reported that transmitochondrial cybrid cell lines from patients with wet AMD had significant disruption of mitochondria functions, increased apoptosis, and cell death along with altered expression levels for complement, inflammation, and angiogenesis genes [11,12,13].

Transmitochondrial cybrids are cell lines that have identical nuclei but mitochondria from different individuals [14]. In another study conducted by Kenney et al., there was a remarkable level of damaged mitochondria in 34 keratoconus corneas versus 33 normal corneas. The relationship between increased oxidative stress and compromised mitochondrial DNA (mtDNA) integrity may play a significant role in this context [15]. As a result, alternative methods of mitochondrial protection might have promising implications in the treatment of some ocular disorders and age-related diseases.

A variety of FDA-approved drugs such as PU-91 are being considered as mitochondria-stabilizing agents with potential therapeutic roles in AMD and other aging diseases where mitochondria are damaged [16]. Our lab previously demonstrated that a lower dose of PU-91 (50μM) had a significant rescuing effect in AMD cybrid cell lines. After 48 and 72 h of PU-91 treatment, PGC-1α (Peroxisome-proliferator-activated receptor Gamma Coactivator-1 alpha), a fundamental regulator of mitochondrial biogenesis, was significantly upregulated [17]. Another study from our lab reported that this low dose of PU-91 increased mitochondrial membrane potential and cellular metabolism while decreasing oxidative mitochondrial injury in AMD cybrid cells with different mitochondrial haplogroups (J, K, and U). As a result, it was concluded that PU-91 could benefit AMD cybrid cell lines with various different mtDNA haplogroups [18].

Quercetin is an antioxidative flavanol derived from the flavonoid group of polyphenols found in many fruits, vegetables, and grains. Quercetin possesses diverse properties including anti-inflammatory, anticarcinogenic, and immune-system-boosting effects, as well as hindering lipid peroxidation and promoting mitochondrial biogenesis [19,20]. The main antioxidant activity of quercetin is associated with cytosolic glutathione, through inhibition of the activity and pathways of signal transduction of this enzyme, which leads to reduced production of ROS [21].

The aim of this study is to investigate the therapeutic potential of higher doses of PU-91, quercetin, or in combination (P+Q) on transmitochondrial cybrid cell lines with various mtDNA haplogroups derived from patients with glaucoma (Glc), keratoconus (KC), and age-related macular degeneration (AMD).

## 2. Materials and Methods

### 2.1. Ethics Statement

The present conducted study on human subjects was performed following the stated principles in the Declaration of Helsinki. Research approval was received from the Institutional Review Board of the University of California (UCI IRB #2003-3131).

### 2.2. Methods of Cybrids Creation and Culture Condition

**Figure 1:**
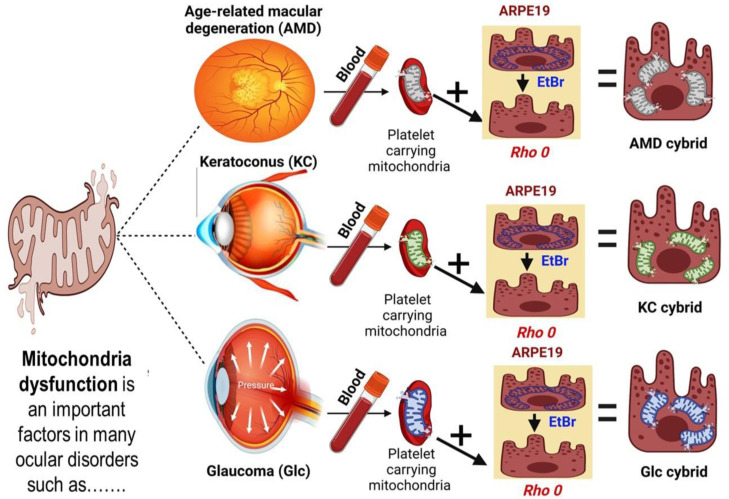
Schematic representation of the preparation of cybrids from the age-related macular degeneration (AMD), keratoconus (KC), and glaucoma (Glc) patients, and age-matched normal (NL) individuals.

The transmitochondrial cybrids were created according to the described process in our previous studies [12] (Figure 1). Tubes with sodium citrate were used for collecting 25 mL of peripheral blood samples. Using DNA extraction kits (Puregene, Qiagen, Valencia, CA, USA), the total DNA was isolated from the white blood cells and quantified with the Nanodrop 1000 (Thermo Scientific, Wilmington, DE, USA). Tris buffer saline (TBS) was used for the isolation of the platelets. In this study, we used ARPE-19 cell lines purchased from ATCC (Manassa, VA, USA) because of similar structural and functional characteristics to in vivo RPE cells [22]. To obtain the ARPE-19 with deficient mtDNA (Rho0), we exposed the cells to low-dose ethidium bromide (50 ng/mL) and then they were five-passaged serially [23]. The DMEM-F12 media contained 50 µg/mL gentamycin, 17.5 mM glucose, 100 µg/mL streptomycin, 10% dialyzed fetal bovine serum, 100 unit/mL penicillin, 2.5 µg/mL fungizone, and 50 µg/mL gentamycin and was used for cell culture media.

A total of 13 cybrid cell lines that contained mitochondria from different individuals with glaucoma (n = 1), keratoconus (n = 3), and AMD (n = 5), along with normal subjects (n = 4), were investigated (Table 1). The passage-5 cybrid cell lines with the confluent conditions were used for all of the performed experiments. Cybrids were cultured in standard culture media alone to select for RPE cells that successfully integrated mitochondria. The mtDNA incorporation was verified using a combination of polymerase chain reaction (PCR) and restriction enzyme digestion of these PCR products. In addition, mtDNA inclusion was verified through mtDNA sequencing to identify the mtDNA haplogroup for each cybrid [24]. In all experiments, cells were treated with PU-91 200 µM (Sigma, LOT# BCCB4083, St. Louis, MO, USA), quercetin 20 µM (GNC, LOT# 4184IU0927, Irvine, CA, USA), and in combination PU-91 with quercetin (P+Q). The main vehicle control was dimethyl sulfoxide (DMSO) (Sigma, St. Louis, MO, USA). In all the experiments, 0.2% of dimethyl sulfoxide (DMSO, Sigma, St. Louis, MO, USA) was used as vehicle control.

### 2.3. Cellular Metabolism Assay (MTT Assay)

In this study, MTT assay was performed for the evaluation of cellular metabolism. Cells were plated in 96-well plates (104/well) and incubated at 37 °C for 24 h. Cells were exposed to either DMSO, PU-91 (200 μM), quercetin (20 μM), or in combination (PU-91+quercetin) for an additional 48 h. Each well received 10 µL of MTT assay reagent (3-(4,5-Dimethyltiazol-2-yl)-2,5-diphenyl tetrazolium bromide; Catalog# 30006, Biotium, CA, USA) and was incubated for 2 h in 37 °C. After adding 100 µL DMSO to each well, plates were read via Biotek Elx808 Absorbance Reader (Winooski, VT, USA).

### 2.4. Intracellular Level of Reactive Oxygen Species (ROS Assay)

The cells were seeded in 96-well plates (104/well). After incubating at 37 °C for 24 h, cells were treated for 48 h with PU-91, quercetin, in combination (PU-91+Quercetin), or DMSO. Then, 100 µL/well H2DCFDA solution (2′, 7′-dichlordihydrofluorescin diacetates; Catalog# D399, Thermo Fisher Scientific, Waltham, MA, USA) was added and plates were analyzed. The fluorescent plate reader measures the excitation (492 nm) and emission (520 nm) wavelengths (SoftMax Pro, version 6.4, Catalog# 94089, Sunnyvale, CA, USA).

### 2.5. RNA Isolation Process and cDNA Amplification

Six-well plates were used for culturing cybrid cell lines. Following 48 **h** of treatment with PU-91, quercetin, (PU-91+quercetin), or DMSO, PureLink RNA Mini Extraction kit (ThermoFisher, Carlsbad, CA, USA) was used for isolating RNA from the cell lysate. RNA quantification was performed using the NanoDrop 1000 (Thermo Scientific, Wilmington, DE, USA). The Superscript IV VILO Master Mix with the Dnase Enzyme (ThermoFisher, Waltham, MA, USA) was used for the reverse transcription of RNA and the creation of complementary DNA (cDNA).

### 2.6. Quantitative Real-Time Polymerase Chain Reaction (qRT-PCR)

The total RNA of cultured cybrid cells treated with PU-91, quercetin, (PU-91+quercetin), and DMSO was isolated. The information of all target primers, which are predesigned via Qiagen QuantiTect Primer Assays or KiCqStart SYBR^®^ Green primers (Sigma–Aldrich, Burlington, MA, USA), is demonstrated in Supplementary Table S1. The qRT-PCR was performed to assess the relative expression levels of genes associated with apoptosis (*BAX* and *CASP3*), inflammation (*IL6*), mitochondrial biogenesis regulators (*TFAM*, *NRF1*, and *PGC1α*), antioxidant enzyme (*SOD2*), and senescence (*CDKN1A* and *LMNB1*) pathways. *HPRT1*, a recycling enzyme of inosine and guanine in the purine salvage pathway, was selected as the housekeeping gene. Therefore, as a stable endogenous control gene, the *HPRT1* primer was considered the reference gene for reaching standard expression levels for all primers. For analyzing the obtained data, the ΔΔCt method was used, in which ΔCt = [Ct (threshold value) of the target gene] − [Ct for HPRT1]; and ΔΔCt = ΔCt of the treatment condition − ΔCt of the untreated condition. For the comparison between untreated conditions versus treated conditions, the fold changes were measured as follows: fold change = 2^−ΔΔCt^. Triplicate formats of treated cells (PU-91, quercetin, and PU-91+quercetin) compared to vehicle-control (DMSO) samples were analyzed.

### 2.7. Statistical Analyses

For statistical analyses, GraphPad Prism (Version 9.1.3, GraphPad Software, Inc., San Diego, CA, USA) was used. Regarding the evaluation of differences among vehicle-control (DMSO) and treated (PU-91, quercetin, PU-91+quercetin), the ANOVA–Kruskal–Wallis test using the two-stage step-up method of Benjamini, Krieger, and Yekutieli by controlling false discovery rate was performed. * Indicates *p* ≤ 0.033; ** ≤ 0.002; *** ≤ 0.0002; and **** ≤ 0.0001 were considered statistically significant.

## 3. Results

### 3.1. Effect of PU-91 (P), Quercetin (Q), or in Combination on Cellular Metabolism and Mitochondrial Biogenesis in Cybrids Derived from Patients with Age Macular Degeneration (AMD), Keratoconus (KC), and Glaucoma (Glc)

**Figure 2:**
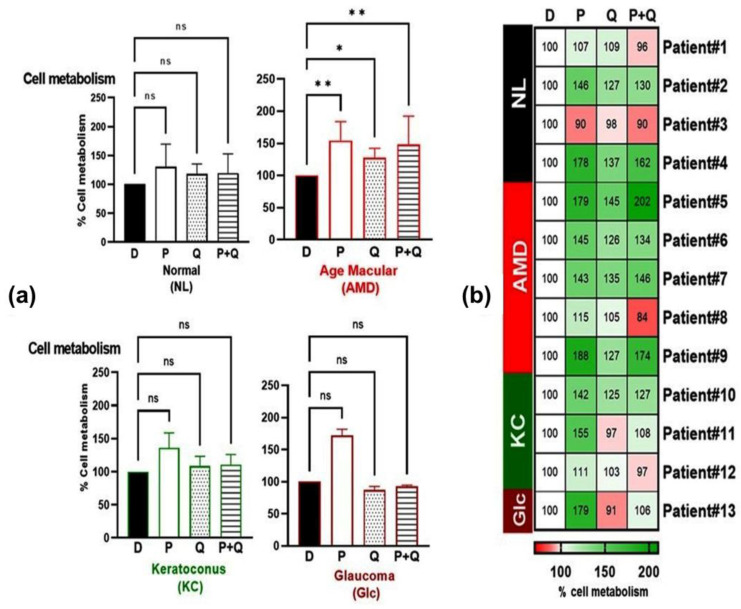
(**a**) Effect of PU-91, quercetin, and in combination on cellular metabolism of normal (NL), age-related macular degeneration (AMD), keratoconus (KC), and glaucoma (Glc) cybrids via MTT assay. MTT assay is used to measure cellular metabolism and cell viability. (**b**) Heatmap representation of the impact of PU-91, quercetin, and in combination on cellular metabolism in patients of normal (NL), age-related macular degeneration (AMD), keratoconus (KC), and glaucoma (Glc) cybrids. * Indicates *p* ≤ 0.033; ** ≤ 0.002, and ns means nonsignificant. In the heatmap representation, a percentage of more than 100% represents higher metabolic activity, indicating higher cell viability.

Our previous published studies showed that treatment with 50 µM concentration of PU-91 improves cellular metabolism oxidative stress and mitochondrial health in AMD cybrids regardless of mtDNA haplogroup (H, U, J, and K) variations [17,18]. These findings prompted us to investigate whether a higher dose (200 µM) of PU-91 would produce the same or better response than the lower dose of PU-91 in cells with mitochondrial dysfunction, such as found in individuals with AMD, keratoconus (KC), and glaucoma (Glc). Our results in Figure 2a show that treatment with PU-91 200 µM increased cellular metabolism by 54% in AMD cybrid (*p*-value = 0.002). The Glc and KC cybrids showed a nonsignificant trend of increased cell metabolism. Previously, we showed that when an esterase inhibitor (EI-12) was combined with PU-91, the positive benefits of PU-91 were maintained by increased cellular metabolism, higher levels of PGC-1α, and reduced apoptosis genes [24]. In the present study, we wanted to determine whether PU-91 combined with another esterase inhibitor, quercetin, had any effect on the cellular metabolism and mitochondrial biogenesis of cybrids derived from subjects with different eye diseases. Interestingly, quercetin alone (*p*-value = 0.02) and in combination (P+Q) treatment (*p*-value = 0.001) increased cellular metabolism in AMD cybrids but not in KC, Glc, or Nl cybrids. Figure 2b shows the heatmap representation for the responses of the individual cybrids within each group (NL, AMD, KC, and Glc). There was great variability in differential cellular metabolism response toward the PU-91, quercetin alone, or in combination (P+Q) within each disease group, representing the personalized responses due to each individual’s mitochondria influence. In the heatmap (Figure 2b), a percentage exceeding 100 indicates cells that are highly metabolically active, which represent higher viability.

**Figure 3:**
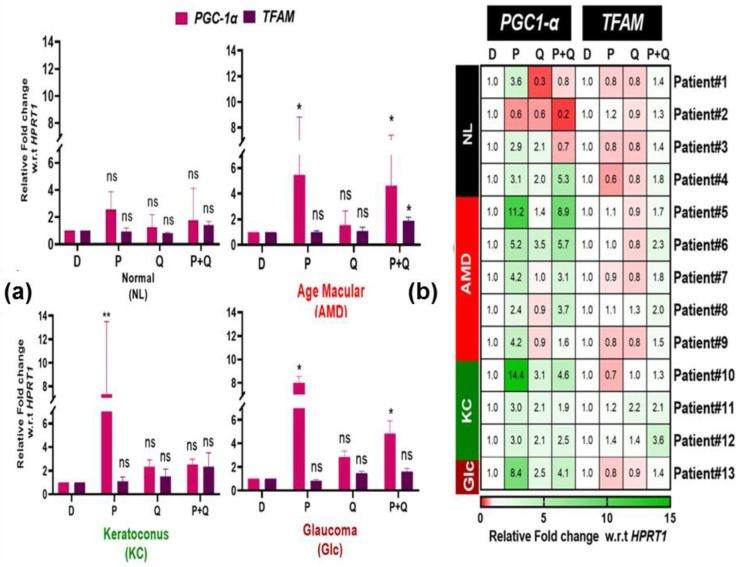
(**a**) Effect of PU-91, quercetin, and in combination on the mRNA expression of PGC-1α and TFAM genes in normal (NL), age-related macular degeneration (AMD), keratoconus (KC), and glaucoma (Glc) cybrids via qPCR. (**b**) Heatmap representation of the impact of PU-91, quercetin, and in combination on the mRNA expression of *PGC-1α* and *TFAM* genes in patients of normal (NL), age-related macular degeneration (AMD), keratoconus (KC), and glaucoma (Glc) cybrids. * Indicates *p* ≤ 0.033; ** ≤ 0.002, and ns means nonsignificant.

Next, we determined whether PU-91, quercetin alone, or a combination (P+Q) increased the expression of genes that regulate mitochondrial biogenesis, such as PGC-1α and TFAM. Our findings show that PGC-1α expression levels had a trend to increase in response to PU-91 treatment in all cybrid groups but achieved statistical significance only in AMD (5.4-fold, ±1.51, *p*-value = 0.015) and Glc cybrids (8-fold, ±0.56, *p*-value = 0.0014) (Figure 3a). Surprisingly, in combination (P+Q) treatment significantly increases PGC-1α expression levels in the Glc cybrids (4.85-fold, ±0.75, *p*-value = 0.032), while it increases expression levels in AMD cybrids but did not reach a statistically significant level (Figure 3a).

The heatmap representation (Figure 3b), demonstrated that the *PGC-1α* expression levels varied from 2.4-fold to 11.2-fold upregulation in the AMD cybrids, indicating that the responses to PU-91 are influenced by the mitochondrial status since each cybrid represents personalized mitochondria from different individuals while the nuclear genome of the cybrids is identical. The KC cybrids showed widely disparate responses to PU-91 with patient #10 showing a 14.4-fold increase in *PGC-1α* expression, while the other two KC cybrids (patient #11 and patient #12) had a threefold expression increase. While there was a slight increase in the expression levels of *PGC-1α* in response to quercetin treatment, it is not statistically significant in NL, AMD, KC, and Glc cybrids.

Increased *TFAM* expression levels were statistically significant in AMD cybrids (1.86-fold, ±0.14, *p*-value = 0.001) in response to the combination treatment (P+Q) (Figure 3a). There was no significant change in *TFAM* expression levels in either of the cybrids in response to PU-91 or quercetin treatment. Similar to the variation of *PGC-1α* expression within each group’s individual cybrids in response to treatment with PU-91, the heatmap showed that *TFAM* expression levels varied to a lesser degree in response to all treatments (Figure 3b).

### 3.2. Effect of PU-91 (P), Quercetin (Q), or in Combination on Reactive Oxygen Species (ROS) and Redox-Sensitive Transcription Factor (NRF1, SOD2) Expression in Cybrids Derived from Patients with AMD, KC, and Glc

**Figure 4:**
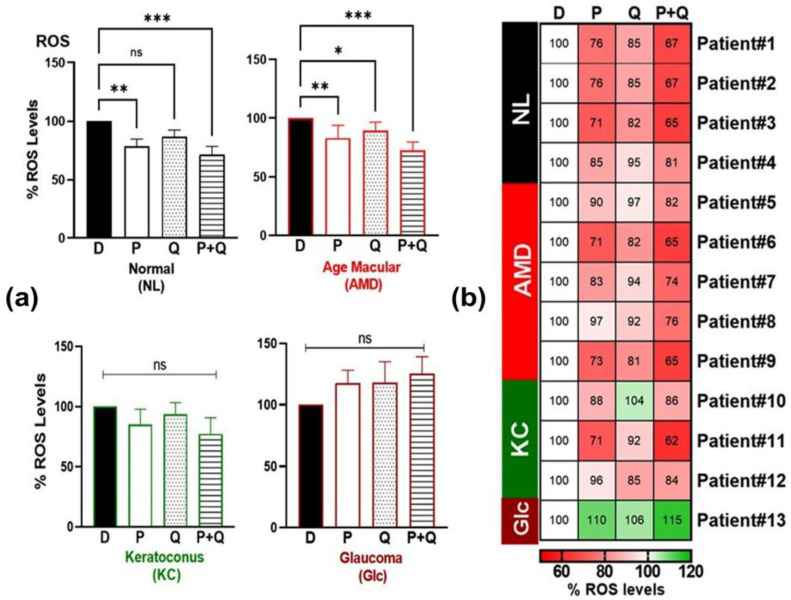
(**a**) Effect of PU-91, quercetin, and in combination on reactive oxygen species (ROS) of normal (NL), age-related macular degeneration (AMD), keratoconus (KC), and glaucoma (Glc) cybrids via ROS assay. (**b**) Heatmap representation of the impact of PU-91, quercetin, and in combination on cellular viability in patients of normal (NL), age-related macular degeneration (AMD), keratoconus (KC), and glaucoma (Glc) cybrids. * Indicates *p* ≤ 0.033; ** ≤ 0.002; *** ≤ 0.0002, and ns means nonsignificant.

Mitochondria are a major source of cellular ROS production [25]. We determined whether PU-91 (P), quercetin (Q) alone, or their combination (P+Q) reduced ROS levels in the NL, AMD, KC, and Glc cybrids. Our results showed that treatment with PU-91 (P), quercetin (Q), and in combination (P+Q) significantly decreased the levels of ROS in AMD cybrid: 82% ± 3.25 (*p*-value = 0.002), 90% ± 3.21 (*p*-value = 0.014), and 72% ± 3.29 (*p*-value = 0.0002), respectively (Figure 4a). The NL cybrids also showed reduced ROS levels after treatment with PU-91 (78.5% ± 6.24, *p*-value = 0.0016) and P+Q treatment (71.2% ± 7.14, *p*-value = 0.0002) but no changes were seen in the KC or Glc cybrids. The heatmap showed individual variability in decreased ROS levels within the AMD and KC disease groups in response to PU-91 (P) or in combination (P+Q) treatments (Figure 4b).

**Figure 5:**
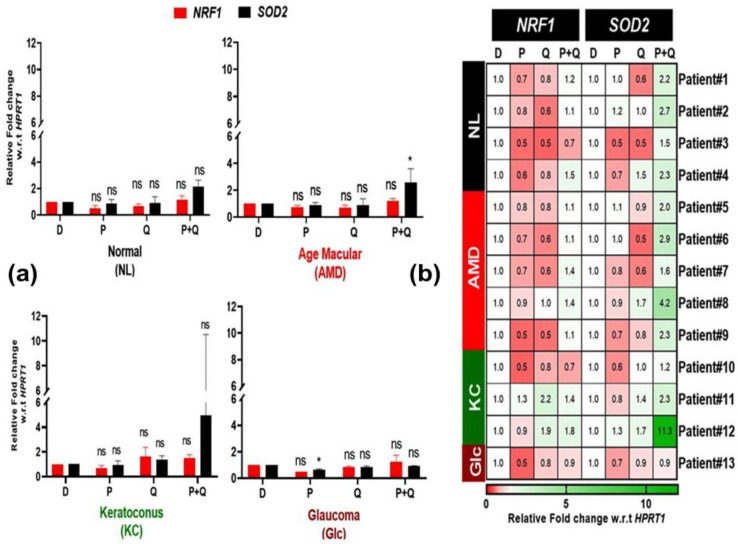
(**a**) Effect of PU-91, quercetin, and in combination on the mRNA expression of antioxidant genes such as *NRF1* and *SOD2* in normal (NL), age-related macular degeneration (AMD), keratoconus (KC), and glaucoma (Glc) cybrids via qPCR. (**b**) Heatmap representation of the impact of PU-91, quercetin, and in combination on the mRNA expression of *NRF1* and *SOD2* genes in patients of normal (NL), age-related macular degeneration (AMD), keratoconus (KC), and glaucoma (Glc) cybrids. * Indicates *p* ≤ 0.033, and ns means nonsignificant.

One important gene that is activated in response to oxidative stress is known as a redox-sensitive transcription factor (*NRF1*), which orchestrates a defense mechanism against ROS-induced cytotoxicity by inducing cytoprotective molecules [26]. As a result, we examined if the reductions in ROS levels in AMD and normal cybrids were related to *NRF1* expression in response to PU-91 (P), quercetin (Q), or both (P+Q). Surprisingly, treatment with PU-91 (P) or quercetin (Q) showed a nonsignificant trend of decreased NRF1 expression levels in AMD and Glc cybrids (Figure 5a). The combination treatment (P+Q) did not significantly alter *NRF1* expression levels in any disease group. Furthermore, the heatmap showed limited variation in *NRF1* expression levels in response to any treatment, with a range from a high value of 2.2 in response to Q treatment (KC patient #11) to a low value of 0.5 (KC patient#10) in response to PU-91 treatment (Figure 5b).

*SOD2*, another important antioxidant gene, is required for the proper functioning of the retinal pigment epithelium (RPE). *SOD2* deficiency causes extensive oxidative damage in the RPE and has been linked to AMD pathogenesis [27]. Our results show that while *SOD2* expression decreases with PU-91 (P) treatment in Glc cybrids (0.63-fold ± 0.06, *p*-value = 0.02), it increases with the combination (P+Q) treatment in AMD cybrids (2.58-fold ± 0.45, *p*-value = 0.031) (Figure 5a). We found the greatest variation in SOD2 expression levels in the KC group’s different individual cybrids in response to combination (P+Q) treatments (Patient #12, high-value range 11.3; and Patient #10, low of 1.2) but not with PU-91 (P) or quercetin (Q) (Figure 5b).

These results suggest that the reduction in ROS levels caused by PU-91 (P) treatment in the AMD cybrid might be unrelated to *NRF1* or *SOD2* expression. In addition, the reduction in ROS levels in response to in combination (P+Q) treatment might be dependent on *SOD2* overexpression but not *NRF1* expression in AMD cybrids. Moreover, the increase in ROS production in the Glc cybrid with PU-91 (P) might be related to *SOD2* lower expression levels.

### 3.3. Effect of PU-91 (P), QUERCETIN (Q), or in Combination on the Expression of Apoptotic Genes in the Cybrids Derived from Patients with AMD, KC, and Glc

**Figure 6:**
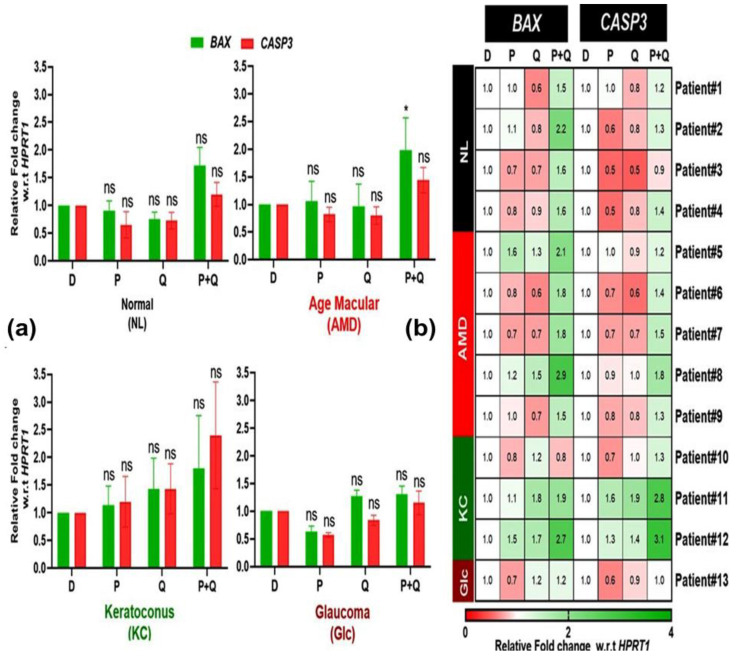
(**a**) Effect of PU-91, quercetin, and in combination on the mRNA expression of *BAX* and *CASP3* genes in normal (NL), age-related macular degeneration (AMD), keratoconus (KC), and glaucoma (Glc) cybrids via qPCR. (**b**) Heatmap representation of the impact of PU- 91, quercetin, and in combination on the mRNA expression of *BAX* and *CASP3* genes in the patients of normal (NL), age-related macular degeneration (AMD), keratoconus (KC), and glaucoma (Glc) cybrids. * Indicates *p* ≤ 0.033, and ns means nonsignificant.

We previously demonstrated that dysfunctional mitochondria in AMD cybrids contribute to increased expression of *CASP3* and *BAX*, both of which are markers of cell apoptosis, but 50 µM PU-91 reversed this upregulation [28]. In this study, we want to determine whether the higher dose of 200 µM PU-91 (P), quercetin (Q), or in combination (P+Q) affects the expression of these apoptotic genes in NL, AMD, KC, and Glc cybrids. Surprisingly, there was a significant increase in the expression of the *BAX* gene in the (P+Q)-treated AMD cybrids and no significant changes in expression levels of BAX or CASP3 after treatment with 200 µM PU-91 or Q alone (Figure 6a). In response to PU-91, the heatmap demonstrated variability in the expression of *BAX* (e.g., AMD Patient #5, 1.6-fold increase and Patient #7, 0.7-fold decrease) and *CASP3* (Patient #11, 1.6-fold increase and Patient #10, 0.7-fold decrease) (Figure 6b).

These findings suggest that neither 200 µM PU-91 nor quercetin was effective in reducing apoptotic gene expression levels. Moreover, the combination (P+Q) treatment promotes apoptosis in AMD cybrids.

### 3.4. Effect of PU-91 (P), Quercetin (Q), or in Combination on the Expression of the Inflammatory Gene in the Cybrid Derived from Patients with AMD, KC, and Glc

**Figure 7:**
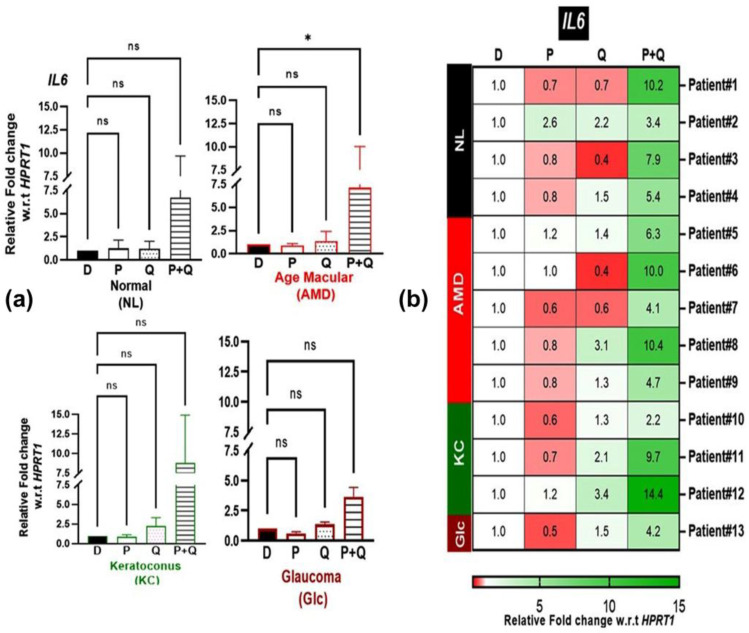
(**a**) Effect of PU-91, quercetin, and in combination on the mRNA expression of *IL6* gene in normal (NL), age-related macular degeneration (AMD), keratoconus (KC), and glaucoma (Glc) cybrids via qPCR. (**b**) Heatmap representation of the impact of PU-91, quercetin, and in combination on the mRNA expression of *IL6* gene in the patients of normal (NL), age-related macular degeneration (AMD), keratoconus (KC), and glaucoma (Glc) cybrids. * Indicates *p* ≤ 0.033, and ns means nonsignificant.

Our previous studies have shown that 50 µM PU-91 reduced inflammation markers in haplogroup H AMD cybrids [17]. This prompted us to investigate whether 200 µM PU-91 (P), quercetin (Q), or a combination of the two (P+Q) would affect the expression of *IL6*, a marker of inflammation, in the cybrid groups. The in combination (P+Q) treatment resulted in significantly increased expression of the *IL6* gene only in AMD cybrids (*p*-value = 0.018) (Figure 7a). Furthermore, we observed more pronounced differences in *IL6* expression when the individual cybrids were treated with P+Q combination compared to PU-91 alone or quercetin alone (Figure 7b).

These findings show that the combination of P+Q induces significant upregulation of *IL6*, a proinflammatory in NL, AMD, KC, and Glc cybrids, making this combination likely harmful to the cell health. However, when the 200 µM PU-91 alone was administered, then the results showed no significant increase in *IL6* expression (Figure 7a), but when viewed individually (heatmap) (Figure 7b), there was variability in the responses (patient #13, 0.5-fold decline, while patient #2 showed 2.6-fold increase). This type of variability of responses is consistent with our previous studies of AMD cybrids that had different mtDNA haplogroups [18,24].

### 3.5. Effect of PU-91 (P), Quercetin (Q), or in Combination on the Expression of Genes Associated with Senescence in the Cybrids Derived from Patients with AMD, KC, and Glc

**Figure 8:**
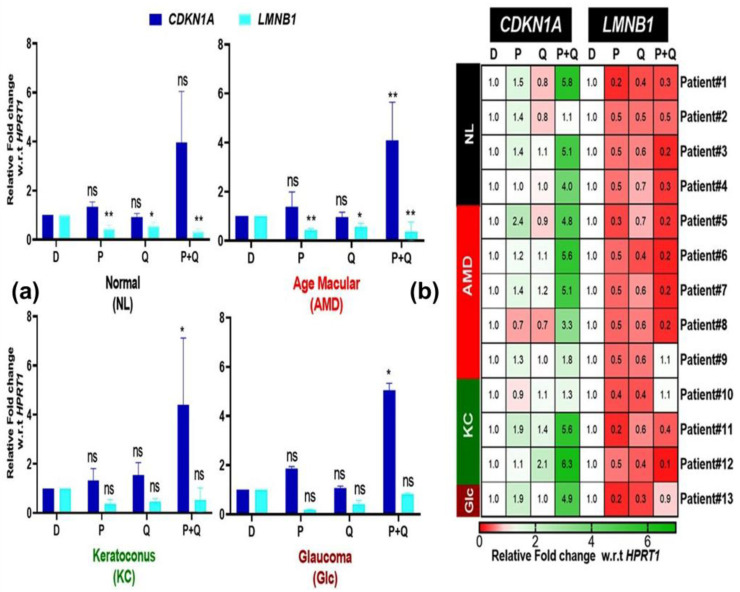
(**a**) Effect of PU-91, quercetin, and in combination on the mRNA expression of *CDKN1A* and *LMNB1* genes in normal (NL), age-related macular degeneration (AMD), keratoconus (KC), and glaucoma (Glc) cybrids via qPCR. (**b**) Heatmap representation of the impact of PU-91, quercetin, and in combination on the mRNA expression of *CDKN1A* and *LMNB1* genes in the patients of normal (NL), age-related macular degeneration (AMD), keratoconus (KC), and glaucoma (Glc) cybrids. * Indicates *p* ≤ 0.033; ** ≤ 0.002, and ns means nonsignificant.

The upregulation of *CDKN1A* and downregulation of *LMNB1* are senescence genes linked to aging [29]. We investigated whether treatment with PU-91 (P), quercetin (Q), or in combination (P+Q) would increase/decrease the expression of *CDKN1A* and *LMNB1* in NL, AMD, KC, and Glc cybrids. The CDKN1A levels were not changed in response to PU-91 or Quercetin (Figure 8a). Surprisingly, in combination (P+Q) treatment significantly increases *CDKN1A* expression in AMD (4.09-fold ± 0.69, *p*-value = 0.001), Glc cybrids (5.05-fold ± 0.20, *p*-value = 0.014), and KC cybrids (4.41-fold ± 2.71, *p*-value = 0.027) (Figure 8a). *LMNB1* expression was downregulated in AMD cybrids with PU-91 (P) (0.44-fold ± 0.06, *p*-value = 0.001), quercetin (Q) (0.57-fold ±0.14, *p*-value = 0.021), and in combination (P+Q) (0.37-fold ± 0.39, *p*-value = 0.002) treatment (Figure 8a). Furthermore, the heatmap showed that the expression levels of *CDKN1A* and *LMNB1* were variable in the normal, AMD, and KC cybrids in response to in combination (P+Q) compared to PU-91 (P) alone or quercetin (Q) alone treatments (Figure 8b). These findings suggest that in combination (P+Q) treatment activates the genes related to the senescence pathway more than PU-91 (P) or quercetin (Q) treatment alone.

## 4. Discussion

**Figure 9:**
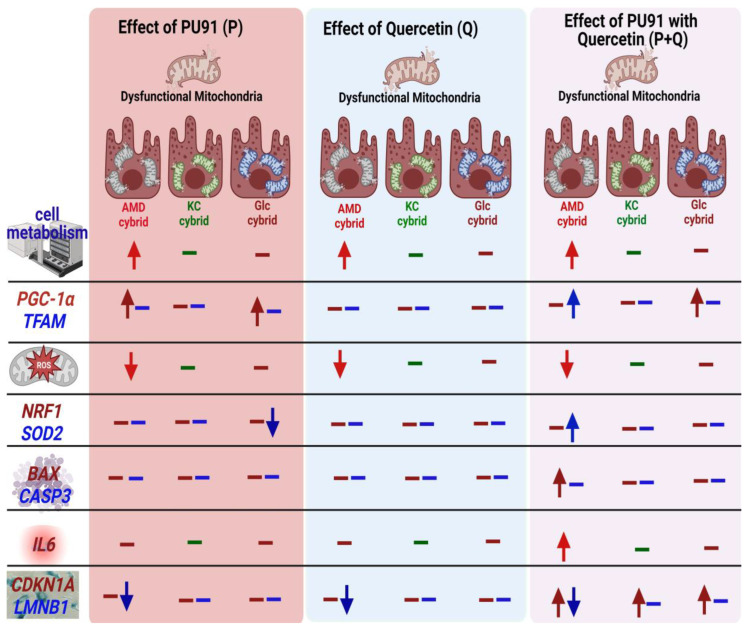
Schematic representation of the effect of PU-91 (P), quercetin (Q), and in combination (P+Q) on the age-related macular degeneration (AMD), keratoconus (KC), and glaucoma (Glc) on the cellular metabolism, ROS levels, expression of genes related to mitochondrial biogenesis, antioxidant genes, apoptotic genes, inflammatory gene, and senescence genes.

In this study, we showed that 200 µM PU-91 (P), quercetin (Q) alone, or in combination (P+Q) had different effects on cellular metabolism, ROS, and genes related to apoptosis, antioxidation, inflammation, and senescence in cybrids generated from patients with different eye diseases (Figure 9). Most importantly, there was considerable interindividual variability in cybrids even within the cybrids of the same disease type. In addition, we found that in combination (P+Q) treatment has no significant beneficial effects in any cybrids, regardless of disease type. Although it increased cellular metabolism and decreased ROS levels in AMD cybrids, this treatment increased levels of *IL6* and *CASP3* in AMD cybrids and modulated senescence-related genes (*CDKN1A* and *LMNB1*) in AMD, KC, and GLc cybrids.

Our findings showed that the 200 µM dose of PU-91 (P) had beneficial effects on the AMD and glaucoma cybrids. Nashine et al. [17] previously demonstrated that 50 µM PU-91(P) regulates the mitochondrial biogenesis pathway, improves cellular metabolism, and prevents apoptotic cell death, ROS production, and inflammation. While 200 µM PU-91 (P) increased cellular metabolism and upregulation of *PGC1α* along with decreased ROS production in AMD cybrids, it also did not have a significant effect on apoptotic cell death or inflammation genes. This demonstrates that treatment with 50 μM PU-91 was more beneficial than treatment with 200 μM PU-91 in the cybrid model.

Mitochondrial DNA (mtDNA) plays a role in mitochondrial function, and changes in mtDNA content, integrity, and transcript level may influence the generation of ROS and play a role in the pathogenesis of AMD, Glc, and KC [15,30,31,32,33,34,35,36]. Our previous studies demonstrated that 50 µM PU-91 preserved the function and integrity of AMD mitochondria and protected against cell death caused by oxidative stress and mtDNA [17]. The antioxidant, anti-inflammatory, and other activities of quercetin may have an impact on treatments for many ophthalmological diseases [37,38].

Our previous studies showed that co-administering PU-91 (P) with esterase inhibitors (EI-12 or EI-78) did not alter or diminish the positive effects of 50 µM PU-91 on cellular metabolism, mitochondrial biogenesis, apoptosis, or inflammation [17]. One of the novel findings in this study was that 200 µM PU-91 alone promotes cellular metabolism in the AMD cybrids in a manner distinct from quercetin alone or in combination. Furthermore, in AMD and GLc cybrids, PU-91 significantly increases PGC-1α levels, which will improve mitochondrial health. The combination (P+Q) treatment significantly reduced ROS production in AMD cybrids but not in KC and Glc cybrids, which could be explained by the upregulation of antioxidant genes such as SOD2 in the AMD cybrids. In contrast, the KC cybrids showed variable responses to the P+Q combination, with patient #12 showing an 11.3-fold increase of *SOD2,* while the other KC cybrids showed only modest elevations.

One of the intriguing findings in this study was that in AMD cybrids, the in combination (P+Q) treatment increased the expression of apoptotic (*BAX*) and inflammatory (*IL6*) genes, which can negatively affect cellular homeostasis. In contrast, Donaldson et al. (2019) hypothesized that quercetin and fenofibrate are synergistic in lowering cholesterol content in an in vivo study and thereby would have beneficial effects [39]. Our findings suggest that the combination (P+Q) treatment may not be the best option for ocular disorders associated with mitochondrial dysfunction, such as AMD, Glc, and KC.

One of the most important properties of quercetin is that it protects against oxidative stress, aging, inflammation, and mitochondrial damage [38]. In senescence, there is an upregulation of *CDKN1A* and a parallel downregulation of *LMNB1*. In our study, quercetin induced a decrease in the expression of genes associated with senescence (*LMNB1*) in NL and AMD cybrids, while it did not change the levels in KC and Glc cybrids. Zoico et al. (2021) demonstrated that treating senescent adipocytes with quercetin reduces senescence [40]. One of the most intriguing findings in our study was that the combination (P+Q) treatment activated the senescence pathway in AMD, KC, and Glc cybrids. Our findings differ from another study, which observed that when quercetin is combined with fenofibrate, it reduces senescence in osteoarthritis patients [39]. Recalde et al.’s study [41] used 10 and 50 μM fenofibrate concentrations to prevent cartilage degradation and to positively modulate key molecular mechanisms such as senescence. This confirms that a 200 μM dose of PU-91 could be the cause of increased expression of genes involved in the senescence pathway. Moreover, in accordance with our current findings, we hold the belief that the contrasting response to treatments between aged cybrids (AMD) and younger ones (KC) can be attributed to systemic age-related changes.

## 5. Conclusions

In conclusion, a 200 µM dose of PU-91 promotes cellular metabolism by upregulating mitochondrial biogenesis in AMD cybrids in contrast to KC cybrids (Figure 9). When compared to the 50 µM PU-91 used previously [23], the 200 μM had less beneficial effects on the AMD cybrids. In AMD patient-derived cybrids, the combination (P+Q) treatment promotes cellular metabolism and reduces ROS production by promoting mitochondrial biogenesis and increasing the expression of the *SOD2* enzyme gene. However, the P+Q treatment also increases the expression of inflammation (*IL6*) in AMD cybrids, and senescence (*CDKN1A*) genes in all patient-derived cybrids, regardless of their disease type.

## Figures and Tables

**Table 1:** Demographics of the K, U, and J cybrids.

Patient #	Cybrid	Haplogroup	Age (y)	Sex	Ethnicity	Diagnosis
1	17.201	H	77	M	White	NL
2	21.264	H	89	M	White	NL
3	15.150	K1a1b1a	59	M	White	NL
4	19.245	A2e	62	F	White	NL
5	13.128	H7e	86	M	White	Early dry AMD
6	17.199	H	83	M	White	dry AMD
7	19.256	H	86	M	White	dry AMD
8	21.263	H	84	F	White	dry AMD
9	14.139	H17b	81	F	White	wet AMD
10	16.188	K2a2a1	90	M	White	KC
11	18.220	K	78	M	White	KC
12	19.259	H	73	M	White	KC
13	18.241	H	80	M	White	Glc

## Data Availability

All data relevant to the study are included in the article.

## Supplementary Materials

The following supporting information can be downloaded at: https://www.mdpi.com/article/10.3390/antiox12071326/s1.
