# Supplementary material for: Potential Therapeutic Functions of PU-91 and Quercetin in Personalized Cybrids Derived from Patients with Age-Related Macular Degeneration, Keratoconus, and Glaucoma
Source: Antioxidants (Basel). 2023 Jun 22;12(7):1326. doi: 10.3390/antiox12071326 (PMC10375999; doi:10.3390/antiox12071326)
Supplement: Supplementary file 1 [file antioxidants-12-01326-s001.zip › antioxidants-2432522-Supplementary file-highlight.pdf]

antioxidants

MDPI

Supplementary files

Supplementary Table S1. Information on the genes associated with antioxidant enzymes, mitochondrial biogenesis regulators, inflammatory, and apoptotic pathways in cybrid cells

Symbol	Gene Name	GenBank Accession No.	Function
HPRT1	Hypoxanthine Phosphoribosyl transferase 1	NM_000194	The protein encoded by this gene is a transferase, which catalyzes the conversion of hypoxanthine to inosine monophosphate and guanine to guanosine monophosphate via the transfer of the 5-phosphoribosyl group from 5-phosphoribosyl 1-pyrophosphate. This enzyme plays a central role in the generation of purine nucleotides through the purine salvage pathway.
CASP3	Caspase 3, apoptosis-related cysteine peptidase	NM_004346	Encodes protein as a cysteine-aspartic acid protease that plays a central role in the execution phase of cell apoptosis.
BAX	BCL2 associated X, apoptosis regulator	NM_004324	This protein encoded by this gene belongs to the BCL2 protein family. BCL2 family members form hetero- or homodimers and act as anti- or pro-apoptotic regulators that are involved in a wide variety of cellular activities. This protein forms a heterodimer with BCL2 and functions as an apoptotic activator.
IL6	Interleukin 6	NM_000600	This gene encodes a cytokine that functions in inflammation and the maturation of B cells. In addition, the encoded protein has been shown to be an endogenous pyrogen capable of inducing fever in people with autoimmune diseases or infections
SOD2	Superoxide dismutase 2	NM_000636	This gene is a member of the iron/manganese superoxide dismutase family. It encodes a mitochondrial protein that forms a homotetramer and binds one manganese ion per subunit. This protein binds to the superoxide byproducts of oxidative phosphorylation and converts them to hydrogen peroxide and diatomic oxygen.
NRF1	nuclear respiratory factor 1	NM_00104011	This gene encodes a protein that homodimerizes and functions as a transcription factor which activates the expression of some key metabolic genes regulating cellular growth and nuclear genes required for respiration, heme biosynthesis, and mitochondrial DNA transcription and replication.
TFAM	transcription factor A, mitochondrial	NM_003201	This gene encodes a key mitochondrial transcription factor containing two high-mobility group motifs. The encoded protein also functions in mitochondrial DNA replication and repair.
PGC1α	PPARG coactivator 1 alpha	NM_013261	The protein encoded by this gene is a transcriptional coactivator that regulates the genes involved in energy metabolism. This protein interacts with PPARgamma, which permits the interaction of this protein with multiple transcription factors. This protein can interact with, and regulate the activities of, cAMP response element binding protein (CREB) and nuclear respiratory factors (NRFs).
CDKN1A	Cyclin-dependent kinase inhibitor 1A	NM_000389	This gene encodes a potent cyclin-dependent kinase inhibitor. The encoded protein binds to and inhibits the activity of cyclin-cyclin-dependent kinase2 or -cyclin-dependent kinase4 complexes, and thus functions as a regulator of cell cycle progression at G1. The expression of this gene is tightly controlled by the tumor suppressor protein p53, through which this protein mediates the p53-dependent cell cycle G1 phase arrest in response to a variety of stress stimuli.
LMNB1	Lamin B1	NM_00119855 7	This gene encodes one of the two B-type lamin proteins and is a component of the nuclear lamina. Duplication of this gene is associated with autosomal dominant adult-onset leukodystrophy (ADLD). Alternative splicing results in multiple transcript variants.
